# Incidence, Healthcare Resource Use and Costs Associated With Incisional Hernia Repair

**DOI:** 10.3389/jaws.2024.12452

**Published:** 2024-02-28

**Authors:** Laurie Smith, Emily Wilkes, Chris Rolfe, Petra Westlake, Julie Cornish, Paul Brooks, Jared Torkington

**Affiliations:** ^1^ Department of Colorectal Surgery, Cardiff and Vale University Health Board, Cardiff, United Kingdom; ^2^ Real-World Evidence, OPEN Health, Marlow, United Kingdom; ^3^ Market Access, Becton Dickinson Surgery UK, Wokingham, United Kingdom

**Keywords:** incisional hernia, incisional hernia prevention, outcomes, morbidity, incisional hernia repair

## Abstract

**Background:** Incisional hernia (IH) is a common complication of abdominal surgery affecting between 12.8% and 30% of patients. In spite of this, rates of IH repair remain low, at around 5% in the literature. We aimed to assess the rate of IH repair in the UK across surgical specialties and the cost burden associated with IH repair.

**Methods:** This is a retrospective observational study of patients undergoing abdominal surgery in England between 2012 and 2022 using the Hospital Episode Statistics (HES) database. Index abdominal surgery was identified between March 2014 and March 2017. Diagnostic and surgical procedure codes were used to identify pre-operative risk factors, index surgeries, IH repair and healthcare contact. Healthcare resource use (HCRU) costs were derived for index surgery and all post-index, non-elective inpatient admissions and outpatient visits using Healthcare Resource Group (HRG) codes within HES.

**Results:** Of 297,134 patients undergoing abdominal surgery, 5.1% (*n* = 15,138) subsequently underwent incisional hernia repair. By specialty, rates were higher in Colorectal (10.0%), followed by Hepatobiliary (8.2%), Transplant (6.8%), Urological (4.0%), Bariatric (3.5%), Vascular (3.2%) and Gynaecological (2.6%) surgery. Patients undergoing IH repair had more healthcare contacts, longer length of inpatient stays and more A+E visits vs. those with no IH repair post index surgery (83% ≥ 1 A+E visit vs. 69%), as well as higher rates of referral to mental health services (19.8% vs. 11.5%). IH repair was associated with an average HCRU cost of £23,148 compared to £12,321 in patients with no IH repair.

**Conclusion:** Patients undergoing IH repair have a greater morbidity than those not undergoing repair, shown by higher HCRU and more healthcare contacts. Despite this, rates of surgery for IH are low, suggesting that most patients with hernias are not undergoing repair. Emphasis must be placed squarely on primary prevention, rather than cure.

## Introduction

Incisional hernia (IH) is a common complication of abdominal surgery, affecting 12.8%–30% of patients [1, 2]. Risk factors for developing IH include, but are not limited to, increasing age, obesity, smoking, location of surgical incision, suture material and closure technique [1, 3–6]. Extensive research has focussed on prevention of IH and risk reduction strategies, yet despite use of these interventions, incidence of incisional hernia remains at 10%–13% [3, 7, 8].

The impact of incisional hernia to both patients and healthcare services should not be overlooked. Patients with incisional hernia report significantly lower quality of life and body image scores compared to patients without [9]. Furthermore, operative repair of incisional hernia is challenging, carrying high recurrence rates and an overall mortality rate of 1% [10, 11]. Patients undergoing repair of incisional hernia may enter a vicious cycle of recurrence and re-operation with increasingly poor outcomes with each attempt [12]. The cost of this to healthcare services is significant, with hernia-related healthcare expenditure in the United States reaching $3.2 billion dollars annually [12, 13].

Prevention of incisional hernia is of the utmost importance in reducing the associated morbidity and cost of incisional hernia. Recent techniques such as the small stitch technique and mesh prophylaxis have shown promising results, yet widespread uptake both remains low [14]. Attitudes towards abdominal wall closure following abdominal surgery potentially reflect a misunderstanding about the burden of incisional hernia to patients and healthcare services.

This study aims to quantify the incidence of incisional hernia repair following open abdominal surgery and the impact of incisional hernia to patients and healthcare services in England.

## Methods

This was a retrospective observational study using population level data taken from the Hospital Episode Statistics (HES) database. The primary objective of this study was to describe the rate of incisional hernia repair following open abdominal surgery. Secondary objectives include the rate of IH repair according to surgical specialty, identifying risk factors for subsequent IH repair and the healthcare resource usage (HCRU) and cost associated with IH repair.

### HES Database

HES is a data warehouse containing records of all patients admitted to NHS hospitals in England [15]. The HES database contains data on hospital diagnoses, procedures, treatment, healthcare resource use (including inpatient admissions [elective and non-elective], outpatient visits, and accident and emergency [A&E] visits). Associated Healthcare Resource Group (HRG) codes are also recorded in HES to track the activity-based income received by hospitals in England for given HCRU [16].

For this study access to HES was provided under licence via Harvey Walsh Ltd. (operating as OPEN Health Ltd.) from NHS Digital. Data Sharing Agreement: DARS-NIC-05934-M7V9K. Copyright ^©^ 2023, NHS Digital. Re-used with the permission of NHS Digital. All rights reserved. Harvey Walsh Ltd. follow NHS Digital HES Analysis Guidelines and required security policies to ensure that data was handled appropriately.

### Patient Selection: Identification of Index Surgery and Incisional Hernia

This study included patients with an Office of Population Censuses and Surveys Classification of Surgical Operations and Procedures 4th Revision (OPCS-4) code recorded within an inpatient admission for intra-abdominal, urologic or gynaecologic surgery in HES between 1st April 2014 and 31st March 2017. A list of OPCS codes used can be seen in Supplementary Appendix SX. The first such surgery within this period was termed the patient’s “Index Surgery.” Electronic medical records (EMR) in HES between 1st June 2012 and 30th June 2022 were used to identify risk factors at index surgery, comorbidities, post-index HCRU and incisional hernia repair. Patients were excluded if they had less than 12 months follow up, had an incisional hernia repair prior to or during their index surgery, had multiple surgical specialties at the time of index surgery, or who had a caesarean section recorded at any time.

Patients identified as undergoing index surgery were further categorised into two sub-cohorts: Those with incisional hernia repair post-index surgery (termed IH repair); defined by the presence of both an ICD-10 code for abdominal hernia and an OPCS-4 procedure code for IH repair within the same HES inpatient admission (see Supplementary Appendix SX for code list), which was used as a surrogate for incisional hernia, and those who did not fit the above criteria (termed no IH repair).

### Identification of Pre- and Post-Index Surgery Variables

International Statistical Classification of Diseases and Related Health Problems 10th Revision (ICD-10) codes were used to identify relevant pre-operative risk factors recorded in HES at the time of index surgery and post-operative complications. If an IH repair was recorded post index surgery, only post-operative complications recorded prior to the IH repair were included.

HES data was used to identify scheduled and unscheduled secondary care encounters including elective and non-elective inpatient admissions, outpatient visits and A&E attendance. Length of stay (LOS) was calculated as the duration of each unique non-elective inpatient admissions in days.

HCRU costs were derived by mapping HRG codes to Payment by Results (PbR) NHS National Tariff Workbooks of costs for the applicable financial year of resource use [17]. All-cause HCRU costs were defined per patient as the cumulative cost of index surgery, and all post-index inpatient admissions (elective and non-elective) and outpatient visits within the study period.

### Statistical Analysis

Statistical analysis was completed using R version 4.2.2 (R Core Team, 2022). Rates of IH repair were calculated as a percentage of patients undergoing a post-index IH repair over the total population of patients with an index surgery of interest recorded between 1st April 2014 and 31st March 2017.

In addition, separate univariate Cox proportional hazard (PH) models were used to determine the incidence risk of IH repair from index surgery for pre-operative risk factors of interest with hazard ratios (HRs) with 95% confidence intervals (95% CIs) and associated *p*-values presented. Person-years at risk was calculated from index surgery until earliest of first IH repair recorded, patient death, emigration from England or end of the study period (30th June 2022).

Descriptive statistics were used to describe post-index surgery complications and HCRU.

Small number suppression (i.e., events with <7 occurrences are replaced with a “*”) was conducted in line with NHS digital guidance to prevent inadvertent patient identification [18].

## Results

**FIGURE 1:**
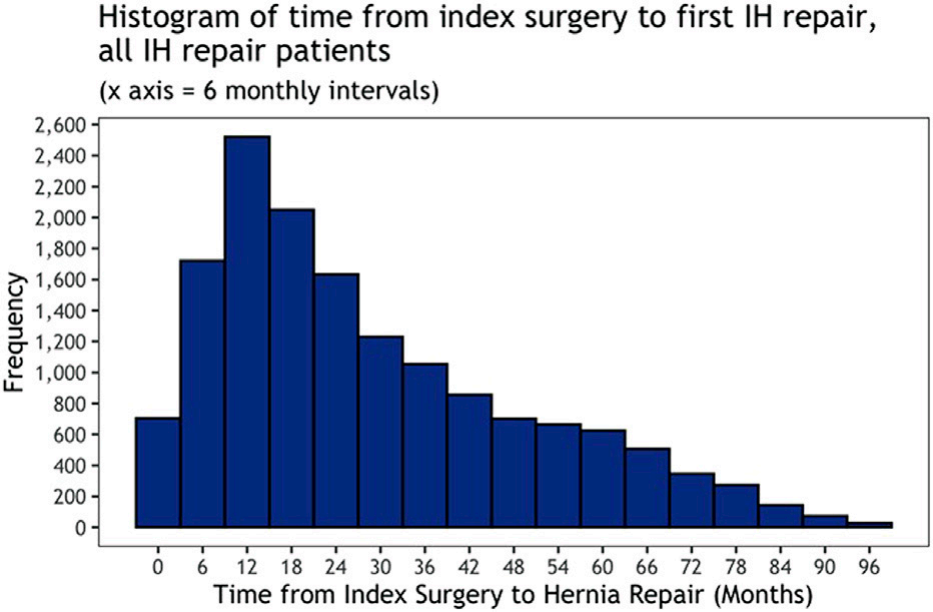
A histogram of time from index surgery to first IH repair for all IH repair patients.

A total of 297,134 patients were included in the study who had undergone abdominal surgery between 1st April 2014 and 31st March 2017 and were followed up for a median time of 6.5 years following that index surgery. Of those, 5.1% (*n* = 15,138) underwent subsequent operative repair of incisional hernia. There were no recorded instances of patients having prophylactic mesh placement at the time of their index surgery. When analysed by index surgical specialty, colorectal surgery had the highest rates of incisional hernia repair at 10.0%, with gynaecological surgery having the lowest rates at 2.6%. A complete breakdown can be seen in Table 1. The median time from index surgery to incisional hernia repair was 24 months (Interquartile range [IQR] 13.42), patients most commonly underwent IH repair within the first 18 months following index surgery (Figure 1).

**TABLE 1:** Rates of incisional hernia repair by surgical specialty.

Surgical specialty	Total patients with index surgery	Incisional hernia (IH) repair	No incisional hernia (IH) repair
Overall	297,134	15,138 (5.1%)	281,996 (94.9%)
Bariatric and Gastrectomy surgery	18,344	635 (3.5%)	17,709 (96.5%)
Colorectal surgery	68,127	6,778 (10.0%)	61,349 (90.0%)
Gynaecologic surgery	83,268	2,142 (2.6%)	81,126 (97.4%)
Hepatobiliary surgery	9,693	797 (8.2%)	8,896 (91.8%)
Transplant surgery	11,548	781 (6.8%)	10,767 (93.2%)
Urologic surgery	75,663	3,030 (4.0%)	72,633 (96.0%)
Vascular surgery	30,491	975 (3.2%)	29,516 (96.8%)

### Risk Factors for IH Repair

Patients undergoing incisional hernia repair had higher rates of diabetes (15.2% vs. 12.5%), smoking (23.5% vs. 18.1%), Chronic Obstructive Pulmonary Disease (COPD) (8.6% vs. 5.1%) and obesity (18.7% vs. 14.7%) at time of their index surgery when compared to patients who did not have IH repair. Univariate analysis of pre-operative risk factors revealed that patients older at index surgery (>50 years of age) (HR: 1.82, 95% CIs: 1.75–1.89, *p* < 0.001), male (HR: 1.44, 95% CIs: 1.39–1.48, *p* < 0.001) and with COPD (HR: 1.91, 95% CIs: 1.80–2.02, *p* < 0.001) had the greatest risk of requiring subsequent incisional hernia repair (see Table 2 for more information).

**TABLE 2:** Univariate analysis of risk factors for IH repair.

	Hazard ratio	95% CIs	*p*-value
Gender			
Female	Reference	Reference	Reference
Male	1.44	1.39, 1.48	<0.001
Age at index operation			
18–50	Reference	Reference	Reference
51–70	1.82	1.75, 1.89	<0.001
71+	1.83	1.75, 1.92	<0.001
Ethnicity			
White (Caucasian)	Reference	Reference	Reference
Asian	0.74	0.67, 0.81	<0.001
Black or Black British	0.59	0.52, 0.66	<0.001
Other	0.75	0.66, 0.86	<0.001
Not known	0.57	0.53, 0.61	<0.001
Not Recorded	0.29	0.17, 0.49	<0.001
Charlson Comorbidity Index (CCI)			
0	Reference	Reference	Reference
1	0.65	0.41, 1.02	0.061
2+	1.74	1.69, 1.80	<0.001
Comorbidities			
Diabetes^a^	1.31	1.25, 1.37	<0.001
Smoking^a^	1.38	1.33, 1.44	<0.001
Chronic obstructive pulmonary disease [COPD]^a^	1.91	1.80, 2.02	<0.001
Obesity^a^	1.29	1.24, 1.35	<0.001
Immunosuppression^a^	1.26	1.14, 1.40	<0.001

^a^ Reference level = “absence”.

### Post-Operative Complications

Of all patients undergoing abdominal surgery, 32.8% (97,371) experienced a postoperative complication, breakdowns of which can be seen in Table 3. Following index surgery, patients who went on to have incisional hernia repair had experienced higher rates of surgical site infection (23.6% vs. 10.1%), wound dehiscence (7.2% vs. 1.6%), bleeding (necessitating blood transfusion) (13.9% vs. 6.8%), fistulation (2.8% vs. 0.6%), small bowel obstruction (10.2% vs. 4.0%). This translated to a longer median length of stay (LOS) at index surgery (7 days [IQR: 4.13] vs. 3 days [IQR: 2.7]) for patients who would have a future repair. Patients undergoing IH repair had higher rates of referral to mental health services (19.8% vs. 11.5%), and chronic pain services (2.1% vs. 1.0%) in the follow-up period compared to patients who did not have IH repair.

**TABLE 3:** Rates of post-operative complications.

Surgical complications following index surgery	Total patients with index surgery	IH repair (*n* = 15,138)	No IH repair (*n* = 281,996)
All combined	97,371 (32.8%)	8,648 (57.1%)	88,723 (31.5%)
Superficial wound infection	32,125 (10.8%)	3,579 (23.6%)	28,546 (10.1%)
Wound dehiscence	5,714 (1.9%)	1,083 (7.2%)	4,631 (1.6%)
Wound haematoma	10,622 (3.6%)	1,525 (10.1%)	9,097 (3.2%)
Post-operative bleeding^a^	21,269 (7.2%)	2,108 (13.9%)	19,161 (6.8%)
Fistula	2,049 (0.7%)	431 (2.8%)	1,618 (0.6%)
Sepsis	28,898 (9.7%)	2,511 (16.6%)	26,387 (9.4%)
Small bowel obstruction	12,950 (4.4%)	1,539 (10.2%)	11,411 (4.0%)
Depression^b^	35,553 (12.0%)	2,998 (19.8%)	32,555 (11.5%)
Chronic pain^b^	3,175 (1.1%)	321 (2.1%)	2,854 (1.0%)

^a^ Necessitating blood transfusion/use of blood products.

^b^ New diagnosis or referral to secondary services post index surgery and prior to IH, repair (if recorded).

### Healthcare Resource Usage and Costs

Table 4 shows the differing healthcare-related attendances between those undergoing IH repair and those who did not, alongside associated costs. In the period following index surgery, 62.5% of patients with an IH repair had ≥2 non-elective admissions to hospital, with the median number of non-elective admissions being 2 (IQR: 1.5) vs. 37.0% of patients without a post-index IH repair, with the median number of non-elective admissions being 1 (IQR: 0.3) in these patients. The median cumulative LOS per patient for non-elective inpatient admissions was 8 days (IQR: 1, 28) in patients with an IH repair and 1 day (IQR: 0, 10) in patients with no repair post index surgery.

**TABLE 4:** Healthcare resource use and costs.

Healthcare resource use		
	IH repair	No IH repair
Index surgery length of stay (LOS) (Days)	7^a^ (4, 13)	3^a^ (2, 7)
Number of patients with at least 1 non-elective admission	12,296 (81.2%)	160,481 (56.9%)
Number of patients with ≥2 non-elective admissions	9,454 (62.5%)	104,227 (37.0%)
Number of non-elective inpatient admissions per patient	2^a^ (1, 5)	1^a^ (0, 3)
Number of elective inpatient admissions per patient	1^a^ (0, 1)	0^a^ (0, 0)
Total cumulative LOS for non-elective inpatient admissions per patient (Days)	8^a^ (1, 28)	1^a^ (0, 10)
Healthcare Resource Use Costs		
Total Costs, Mean per patient (standard deviation[SD])	IH repair	No IH repair
Admission for index surgery	£5,774.78 (4,114.23)	£4,567.13 (3,718.68)
Non-elective inpatient admissions following index surgery	£10,718.94 (16,613.09)	£5,086.53 (10,680.38)
Outpatient visits	£4,095.12 (3,867.99)	£2,666.84 (3,345.16)
All-cause HCRU costs (cumulative cost of index surgery, and all post-index inpatient admissions (elective and non-elective) and outpatient visits)	£23,147.70 (19,071.64)	£12,320.55 (13,054.75)
Total cost of care for matched cohort size (All-cause HCRU costs x number of IH repairs)	£350,409,883	£186,508,486

^a^ Median (IQR).

**FIGURE 2:**
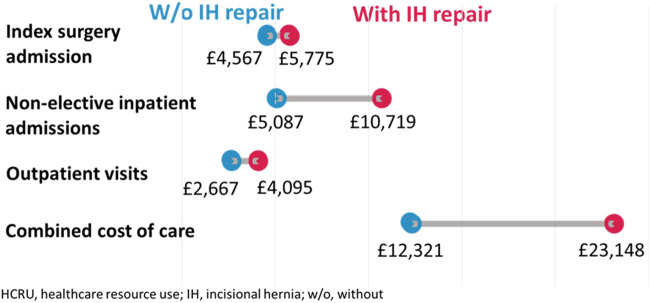
A breakdown of healthcare-associated costs.

Patients who underwent IH repair averaged total costs of £23,147 per patient (pp) in the follow-up period between index surgery and IH repair, compared to £12,320pp in those who did not undergo IH repair (Figure 2). The average cost of an operative IH repair admission was £2,155pp. The total cost of care in patients undergoing IH repair was £350,414,424 compared to £186,515,298 when matching cohort sizes; a cost-difference of £163,899,126. A complete breakdown of healthcare usage and cost can be seen in Table 4.

## Discussion

This retrospective review of population-level data from England describes the post-operative journey that patients undergo following abdominal surgery in England. A recent publication from the French national database estimated the cost of incisional hernia repair to be €4,153 per repair, in keeping with other publications [11]. Our paper is the first, however, to describe the morbidity and cost incurred to patients between their index surgery and repair.

Our results demonstrate that 5% of all patients undergoing abdominal surgery will undergo subsequent repair of incisional hernia. This figure is identical to data published from the French national database by Gignoux et al., who demonstrated a re-operation rate of 5% over a 5-year follow-up period, alongside data from a systematic review by Bosanquet et al which reported the risk of undergoing IH repair of 5.2% [1, 19]. Surgical specialties with higher rates of IH included Colorectal, Hepatobiliary and Transplant surgery, with lower rates identified in Urologic, Bariatric, Vascular and Gynaecologic surgery.

Higher rates of IH repair in colorectal and transplant patients are consistent with the findings of Basta et al., who reported rates of 7.7% and 4.8%, respectively [10]. Hernia repair has traditionally been the bastion of the general surgeon, and higher rates of hernia repair in the general surgical subspecialties may simply be due to increased awareness and early detection, without the delays of referral to another specialty. This is supported by lower-than-expected rates of repair in non-general surgical specialties such as Gynaecology and Urology.

Patients undergoing vascular surgery, specifically aortic surgery, are at increased risk of incisional hernia development, so it is perhaps surprising to see lower rates of incisional hernia repair than average in our cohort [20]. The reasons for this are not immediately apparent but may reflect a more comorbid population group that are unfit for subsequent incisional hernia repair.

In this study, the median time between index surgery and IH repair was 24 months, similar to figured reported by Kockerling et al. in 2015, in which 50% of recurrent incisional hernias had been repaired within 2 years of index surgery [21]. The risk of incisional hernia development continues up to 3 years post-surgery, implying a trend of intervention in early, smaller incisional hernias, rather than chronic complex hernias [22]. Whether this is the case or if this impacts subsequent hernia recurrence rates is beyond the scope of this study.

This paper demonstrates that patients undergoing incisional hernia repair have increased rates of complications after their index surgery, such as surgical site infection, wound dehiscence and fistulation compared to patients that do not undergo repair. Incisional hernia occurs as a result of failure of the abdominal wall to heal. SSI, dehiscence and fistulation represent impaired wound healing and have been recognised in the literature as risk factors for IH development [23–25]. In our cohort, 19.8% of patients undergoing IH repair were referred to mental health services in the time period between index surgery and IH repair. Van Ramshorst et al. in 2012 demonstrated lower quality of life and body image scores in patients with incisional hernia compared to those without, yet there was no difference in scores for mental health between the two groups [9]. The link between IH and referral to mental health services in our results is not clear; the differing rates of in our study may reflect the impact that post-operative complications have on the patient rather than being attributed to the hernia alone. Nonetheless, our data supports the importance of “getting it right first time” both in terms of reducing post-operative complications and subsequent impact on patient wellbeing.

Given that patients undergoing IH repair have higher rates of post-operative complications, it is perhaps not surprising that healthcare associated resource use is higher in this group of patients than in those with no repair. Increased post-operative length of stay, and more unplanned hospital attendances, translates to an average cost difference of £10,827 per patient between each group, and a matched cohort-size cost difference of over £163 million. Consistent with the findings reported in this manuscript, a cost analysis of incisional hernia in a population of US patients published by Fischer et al. demonstrated higher average readmission costs in patients with hernia, as well as higher combined costs of care ($41,053 vs. $81,183, *p* < 0.001) [13]. The costs described in our paper exclude those of the incisional hernia repair and subsequent care and suggest that the financial burden of incisional hernia is greater still.

There are limitations to our study, chiefly the use of incisional hernia repair as a surrogate for diagnosis of incisional hernia. As previously mentioned, rates of incisional hernia vary from 12.8% to 30% in the literature, therefore our rate of repair is an underestimate of the true rate of herniation in our cohort. Further work is needed to identify and chart the morbidity, cost and decision making in patients diagnosed with IH who do not undergo operative repair, as it appears that it is the minority of patients who undergo IH repair. Another limitation is the inability to draw cause and effect between cost, attendances, and IH repair. Our data demonstrates that most of the cost difference is in non-elective admissions. Whether these admissions are related to the incisional hernia itself, or as a consequence of the increased rates of post-operative complications is not clear, and further work in this area is needed. Nonetheless, the risk of needing IH repair is increased in patients who suffer complications following their index surgery. Focus should be on prevention of not just incisional hernia at index surgery, but also on the post-operative complications, such as SSI and wound dehiscence that correlate with increased incisional hernia rates.

This study highlights that 5% of all patients undergoing abdominal surgery will undergo further surgery to repair incisional hernia, and charts what happens to these patients before they undergo their repair. In order to reduce the risk of IH and the burden to both patients and healthcare services, the focus needs to be on prevention. Implementation of current European and American Hernia society guidance, alongside pre-operative risk assessment and targeted mesh-augmented abdominal wall closure will reduce incidence of IH but need to be combined with broader national improvement programmes such as “Getting It Right First Time” (GIRFT) in order to reduce variation in all aspects of post-operative surgical care, across surgical specialties [7, 26]. Prevention of incisional hernia should not be the focus of one surgical specialty and further work is needed to raise awareness of this issue and the issue of prevention outside of the traditional hernia specialties.

## Conclusion

Patients undergoing abdominal surgery are at risk of developing incisional hernia, regardless of surgical specialty. Patients who undergo repair of IH are more likely to have increased rates of post-operative complications and have higher rates of healthcare usage and costs of care compared to patients who do not undergo surgery.

This paper is the first to attempt to map the patient journey between index surgery and first IH repair, but further work is needed to identify the missed majority of patients with incisional hernia who do not undergo repair. Urgent strategies to change practice and implement prevention guidelines are needed to reduce the burden of morbidity and cost associated with IH repair.

## Data Availability

The datasets presented in this article are not readily available as they were retrieved from the Hospital Episodes Statistics (HES) Database; NHS Digital. Information about how to access this database can be found at digital.nhs.uk/data-and-information/data-tools-and-services/data-services/hospital-episode-statistics/users-uses-and-access-to-hospital-episode-statistics.
